# Plasma Interleukin-27 (IL-27) Levels Are Not Modulated in Patients with Chronic HIV-1 Infection

**DOI:** 10.1371/journal.pone.0098989

**Published:** 2014-06-04

**Authors:** Sanjay Swaminathan, Zonghui Hu, Adam W. Rupert, Jeanette M. Higgins, Robin L. Dewar, Randy Stevens, Qian Chen, Catherine A. Rehm, Julia A. Metcalf, Michael W. Baseler, H. Clifford Lane, Tomozumi Imamichi

**Affiliations:** 1 Applied and Developmental Research Directorate, Leidos Biomedical Research, Inc., Frederick National Laboratory for Cancer Research, Frederick, Maryland, United States of America; 2 Department of Clinical Immunology, Western Sydney Local Health District, Sydney, Australia; 3 Sydney Medical School, University of Sydney, Sydney, Australia; 4 School of Medicine, University of Western Sydney, Sydney, Australia; 5 Biostatistics Research Branch, National Institute of Allergy and Infectious Diseases, National Institutes of Health, Bethesda, Maryland, United States of America; 6 Laboratory of Immunoregulation, National Institute of Allergy and Infectious Diseases, National Institutes of Health, Bethesda, Maryland, United States of America; 7 Division of Clinical Research, National Institute of Allergy and Infectious Diseases, National Institutes of Health, Bethesda, Maryland, United States of America; University of South Carolina School of Medicine, United States of America

## Abstract

**Objective:**

IL-27 is an immunomodulatory cytokine with potent anti-HIV properties in PBMCs, CD4+ T cells, macrophages and immature dendritic cells. Previous smaller studies have suggested that HIV-1 infection may alter IL-27 and influence HIV-1 pathogenesis. The aim of this study was to examine the relationship between plasma IL-27 levels in a well-characterised cohort of HIV-1 infected patients.

**Methods:**

Patients were stratified into four groups based on HIV-1 viral load and matched according to age, gender and those receiving antiretroviral treatment. IL-27 levels and C-reactive protein (CRP) were measured using electrochemiluminescence assays. D-dimer and CD4+ T cell counts were measured using an Enzyme Linked Fluorescence Assay and FACS, respectively. sCD14 and sCD163 were measured using ELISA. HIV-1 viral load was measured by bDNA or qRT-PCR assays.

**Results:**

Plasma IL-27 levels were measured in 505 patients (462 HIV+, 43 controls). The mean level (±SEM) of IL-27 in controls was 2990.7±682.1 pg/ml, in the <50 copies/ml group it was 2008.0±274.8 pg/ml, in the 51–10,000 copies group it was 1468.7±172.3 pg/ml, in the 10,001–100,000 copies/ml group it was 1237.9±127.3 pg/ml and in the >100,000 copies/ml group it was 1590.1±223.7 pg/ml. No statistically significant difference in IL-27 levels between groups were seen. There were no correlations noted between IL-27 and HIV-1 viral load or CD4+ T cell counts. There was a small correlation noted between D-dimer and IL-27 (Spearman r = 0.09, p = 0.03) and sCD163 and IL-27 (Spearman r = 0.12, p = 0.005). No correlation was observed between IL-27 and CRP or sCD14 levels.

**Conclusions:**

This is the largest study examining the levels of plasma IL-27 in HIV-1 infection. While IL-27 levels are not significantly altered in HIV-1 infection compared to uninfected controls there may be a small association between IL-27 and D-dimer levels and IL-27 and sCD163 levels.

## Introduction

Interleukin-27 (IL-27) has emerged as an important immunomodulatory cytokine playing pivotal roles in both innate and adaptive immunity. IL-27 is composed of a p28 subunit and an Epstein-Barr virus induced gene 3 (EBI3) subunit [1] and is produced mostly in antigen presenting cells upon stimulation. Binding of IL-27 to its receptor leads to signaling cascades mainly via the JAK/STAT pathway [2]. IL-27 has predominantly anti-inflammatory properties [3] through its actions on cytokines such as IL-10 and IL-21 but also by acting on various CD4+ T cell subsets such as T regulatory cells (Tregs) and Th17 cells [4].

IL-27 has been demonstrated to exhibit potent anti-HIV properties in PBMCs, CD4+ T cells and monocyte derived macrophages (MDMs) [5]–[8]. More recently, IL-27 has been demonstrated to also have potent anti-HIV properties in immature dendritic cells [9] and therefore IL-27 has been shown to have utility in all major cell types targeted by the HIV-1 virus. This potent anti-HIV activity of IL-27 against all major cell types infected with the virus has given credence to potentially using this cytokine as a novel therapeutic agent against HIV-1 infection [10].

To date, there have been two relatively small studies investigating IL-27 levels in patients with HIV-1 infection. In the first of these studies, there was a small negative correlation between IL-27 levels and HIV-1 viral load, with the authors suggesting that HIV-1 infection may be down-regulating IL-27 to promote its pathogenesis [11]. In the second study, a positive correlation was noted between CD4+ T cell counts and IL-27 levels but an analysis of viral load versus IL-27 was not performed [12]. The aim of our study was to investigate the relationship between plasma IL-27 levels and HIV-1 infection in a large, well characterized cohort of HIV-1 infected patients. The secondary aim was to see if IL-27 levels correlated with markers of inflammation (CRP and D-dimer) or with markers of monocyte activation (sCD14 and sCD163). In the current study, 462 HIV-1 infected patients were stratified into four groups based on HIV-1 viral load and IL-27 levels were compared with 43 uninfected controls. We found no correlation between plasma IL-27 levels and HIV-1 viral load or CD4+ T cell count but did observe a small positive correlation between D-dimer levels and IL-27 and between sCD163 levels and IL-27.

## Materials and Methods

### Samples

All HIV-1 positive participants in this study were enrolled in the following clinical protocols: 01-I-0225, 02-I-0125, 02-I-0202, 04-I-0018, 04-I-0187, 05-CC-0127, 05-CC-0246, 05-I-0065, 06-I-0086, 06-I-0153, 06-I-0197, 07-C-0087, 07-I-0001, 08-I-0221, 09-I-0013, 09-I-0030, 09-I-0108, 10-CC-0200, 10-I-0080, 11-CC-0152, 12-CC-0017, 81-I-0164, 88-I-0172 and 91-I-0140 . These protocols were approved by the National Institute of Allergy and Infectious Diseases (NIAID) Institutional Review Board administered at the NIH Clinical Center in Bethesda, MD. All study participants provided informed written consent prior to blood being drawn. Based upon the magnitude of correlation published in earlier papers, we calculated that approximately 120 patients would be sufficient to detect a Spearman rank correlation of 0.3. The HIV-1 positive patients were stratified into four groups depending on HIV-1 viral load (<50 copies/ml, 51-10,000 copies/ml, 10,001–100,000 copies/ml and >100,000 copies/ml) and were closely matched according to number per group, gender, age and those receiving antiretroviral therapy (ART) (demographics listed in Table 1). Patients not on ART, were treatment naïve.

**Table 1:** Demographics of patients used in study.

Group	<50 copies/ml	51–10,001 copies/ml	10,001–100,000 copies/ml	>100,000 copies/ml	Uninfected Controls	Totals
**Number of patients** 1	116	110	117	119	43	505
**No. of males^2^**	84 (72.4%)	84 (76.4%)	92 (78.6%)	95 (79.8%)	25 (58.1%)	380 (75.2%)
**No. of females^3^**	32 (27.6%)	26 (23.6%)	25 (21.4%)	24 (20.2%)	18 (41.9%)	125 (24.8%)
**Mean Age**	47.9±9.6	42.7±12.4	42.7±10.5	39.2±10.1	42.1±10.9	43.2±11.1
**No. of patients on ART**	34 (29.3%)	26 (23.6%)	24 (20.5%)	32 (26.9%)	N/A	116
**Hepatitis C**	9	0	12	0	0	21
**Hepatitis B**	2	0	5	0	0	7
**Mean Viral Load ±SD** 4	<50	2,085±2,604	33,192±23,627	446,580±842,056	N/A	-
**Viral Load range**	<50	53–,945	10,241–97,690	102,480–7,627,300	N/A	-
**Total CD4+T cell count** 5	770±335	518±259	345±269	112±165	Not measured	-
**CD4+T cell count range**	143–1,814	36–1370	1–1,234	0–841	Not measured	-

1: All HIV-1 positive patients were enrolled in protocols approved by the National Institute NIAID Review Board administered at the NIH.

2&3: Numbers in parentheses indicate percentatges of male or females in each group.

4: The HIV-1 viral load was measured using the bDNA [22] or real-time PCR method [23] Data show means +SD (copies/ml).

5: total CD4+T cell count was performed by flow cytometer and absolute CD4 counts were obtained using a dual platform method using a Sysmex XT2000i hematology analyzer. Data indicate means +SD (cells/µl).

### Detection of Biomarkers

IL-27 and C-reactive protein (CRP) measurements were determined by running EDTA preserved plasma in duplicate on a custom electrochemiluminescence assay (Meso Scale Discovery, Rockville, MD). The threshold of detection for CRP and IL-27 using the electrochemiluminescense assay was 0.008 ng/ml and 78.13 pg/mL, respectively. For the IL-27 Meso assay, antibodies were obtained from R&D Systems, Minneapolis, MN, USA. Due to the presence of the EBI3 subunit in both IL-27 and IL-35, an experiment was performed to determine the cross-reactivity between IL-27 (R&D System) and IL-35 (Enzo Life Sciences, New York, USA) and it was found to be 1.8% (10 ng/ml IL-35 was read as 0.188 ng/ml IL-27). sCD14 levels (R&D Systems, Minneapolis, MN, USA) and sCD163 levels (Aviscera Bioscience Inc., Santa Clara, CA, USA) were determined by running EDTA preserved plasma in duplicates using ELISA kits. D-dimer levels were measured by running 200 µl of plasma with an Enzyme Linked Fluorescence Assay (bioMérieux, Marcy l'Etoile, France). Immunophenotyping of peripheral blood drawn into EDTA was performed according to the manufacturer's instructions, by using a modification of the Centers for Disease Control and Prevention guidelines in a Clinical Laboratory Improvement Act (CLIA)-certified laboratory to enumerate CD4+ T cells. Briefly, 100 µl of whole blood was stained with CD3- Fluorescein isothiocyanate (FITC), CD8- R-Phycoerythrin (PE), CD45- Peridinin-chlorophyll proteins (PerCP), and CD4-allophycocyanin (APC) monoclonal antibodies from BDBiosciences (San Jose, CA) then lysed after staining with Optilyse C (Beckman Coulter, Miami, FL), washed twice, and resuspended in 500 µl of phosphate-buffered saline (Lonza, Walkersville, MD). Samples were analyzed immediately on a Becton Dickinson FacsCanto II flow cytometer (BDBiosciences, San Jose, CA). Lymphocytes were enumerated using CD45 PerCP verus side scatter (SSC) gating. The lymphocyte gate was applied to the CD3 FITC versus CD4 APC histogram to isolate the CD4+ T cells which were CD3+CD4+ cells. Absolute CD4 counts were obtained using a dual platform method using a Sysmex XT2000i hematology analyzer (Sysmex America, Mundelein, Illinois) to obtain the white blood count (WBC 10^3^/µL) and the lymphocyte percentage (10^3^/µL) to calculate the absolute lymphocyte count which was multiplied by the CD3+CD4+ percentage from the BD FacsCanto II to obtain the absolute CD4+ count. A minimum of 5,000 lymphocytes were collected for each sample and analyzed with BD FACSDIVA Software version 6.1.3 (San Jose, CA).

### Statistical analysis

Statistical analysis was performed using Prism 6 for Windows (Graph Pad) and comparisons between groups were performed using the Wilcoxon rank-sum statistic (Mann-Whitney), whilst correlations were calculated using the Spearman's rank correlation.

## Results and Discussion

**Figure 1:**
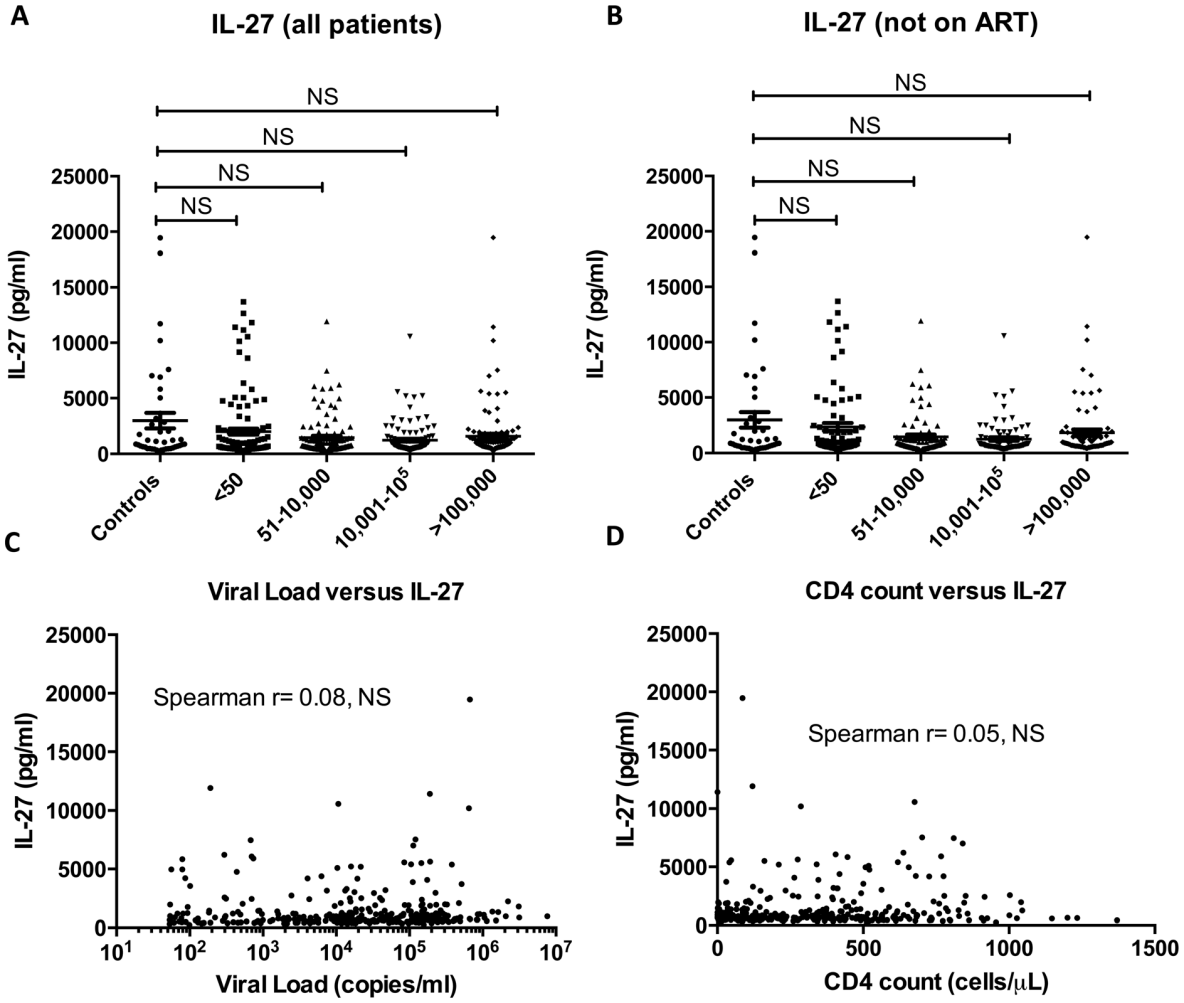
Measurement of plasma IL-27 levels in HIV-1 infected patients and controls. In Panel A, IL-27 levels were measured in plasma from HIV-1 negative controls and also in HIV-1 infected patients divided into 4 groups based on viral load (<50 copies/ml, 51–10,000 copies/ml, 10,001–100,000 copies/ml and >100,000 copies/ml). There were no statistically different differences in plasma IL-27 levels between groups (using non parametric Mann Whitney t statistic). HIV-1 positive patients who were on ART were excluded from the analysis (Panel B) and IL-27 levels were compared between groups which came to the same conclusion. There was no correlation noted between IL-27 levels and viral load (Panel C), nor between IL-27 levels and CD4+ T cell counts (Panel D). Inflammatory markers (D-dimer and CRP) were measured in a subset of patients, which revealed a small correlation between IL-27 and D-dimer (Panel E) but no correlation between CRP and IL-27 levels (Panel F).

IL-27 levels were measured and comparisons were made by stratifying HIV-1 positive patients into four groups according to viral load and comparing these to uninfected controls (Figure 1A). The mean level (±SEM) of IL-27 in the uninfected controls was 2990.7 ± 682.1 pg/ml, whilst in the <50 copies/ml group it was 2008.0±274.8 pg/ml, in the 51-10,000 copies group it was 1468.7±172.3 pg/ml, in the 10,001 – 100,000 copies/ml group it was 1237.9±127.3 pg/ml and in the >100,000 copies/ml group it was 1590.1±223.7 pg/ml. There was no statistically significant difference in levels of IL-27 between groups using the Wilcoxon rank-sum statistic. 25.1% of HIV-1 infected patients in this study were receiving ART and these patients were excluded from analysis in case ART could influence IL-27 levels. Limiting the analysis to the 75% of the cohort that were not on ART, the mean level (±SEM) of IL-27 in the uninfected controls was 2990.7±682.1 pg/ml, whilst in the <50 copies/ml group it was 2362.3±301.0 pg/ml, in the 51-10,000 copies group it was 1476.1±180.6 pg/ml, in the 10,001 – 100,000 copies/ml group it was 1269.1±132.3 pg/ml and in the >100,000 copies/ml group it was 1836.0±168.3 pg/ml. There were no statistically significant differences in IL-27 levels observed between any of the groups stratified by viral load (Figure 1B). There were no differences in IL-27 levels based on gender or in mono-infected patients (without Hepatitis B or C) between groups sorted by viral load either (data not shown). IL-27 levels were also analyzed between those patients on ART versus those who were not on ART in the various HIV+ groups. Interestingly, in the <50 copies/ml group, there was a statistically significant decrease in IL-27 levels in the patients receiving ART versus those who were not on ART (p = 0.0004). None of the other HIV+ groups showed a significant difference in IL-27 levels between patients on ART versus those who were not on ART.

Next, we determined if IL-27 levels were correlated with either HIV-1 viral load or CD4+ T cell count in the HIV-1 positive patients. Firstly, the relationship between HIV-1 viremic patients (those with a viral load of >50 copies/ml, n = 346) with IL-27 levels (Figure 1C) was examined. The Spearman rank correlation (r = 0.08) indicated no statistically significant correlation between these parameters. This is in contrast to the weak negative correlation observed previously [11]. We next determined if there was a correlation between CD4+ T cell counts and IL-27 levels in all HIV-1 positive patients (n = 462) and found no statistically significant correlation (Spearman r = 0.05). A previous report demonstrated a positive correlation between IL-27 and CD4+ T cell count [12] which was not borne out with this study.

**Figure 2:**
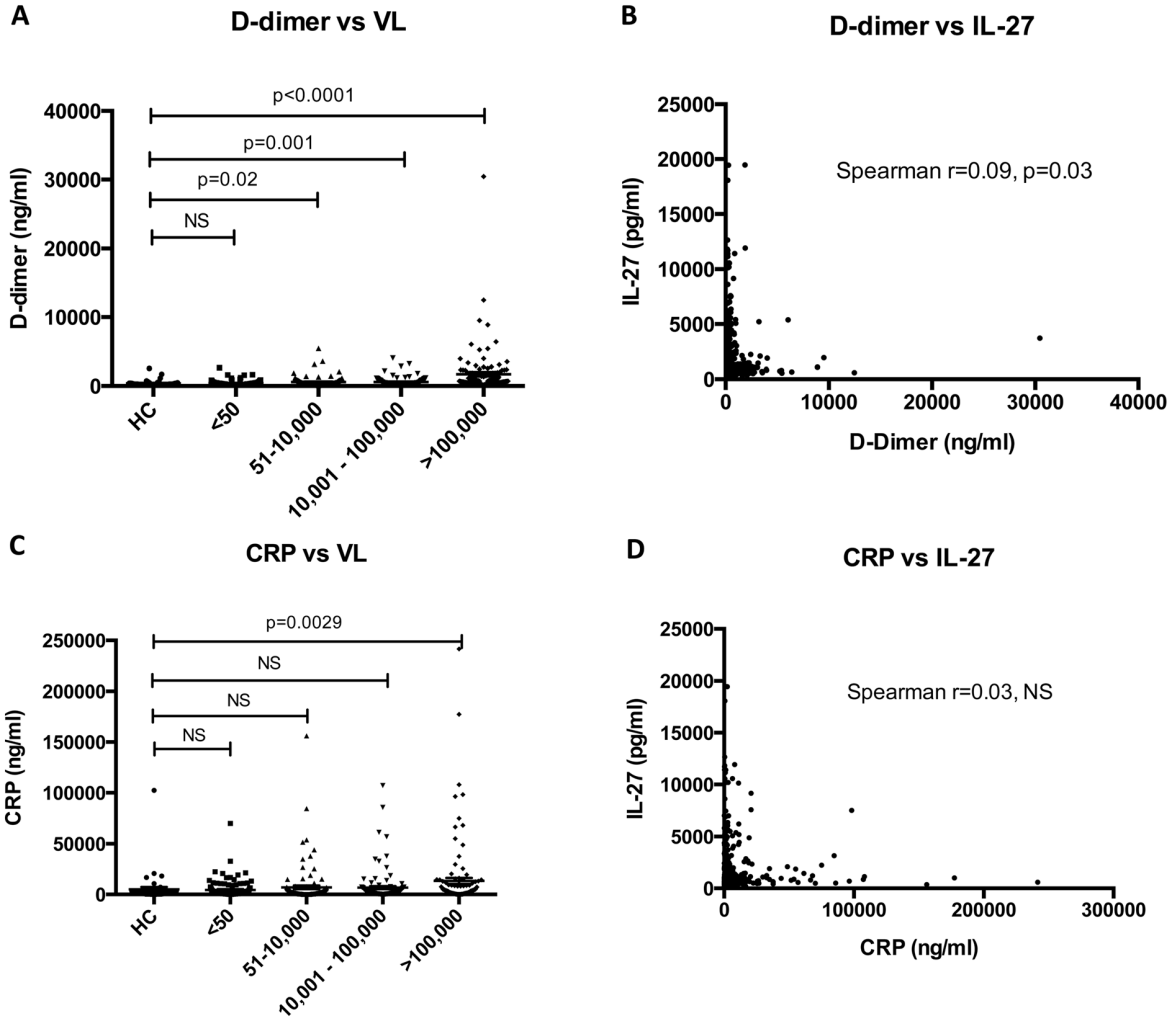
Correlation of D-dimer and CRP with plasma IL-27 levels. In Panel A, D-dimer levels were plotted for each group of patients (stratified by viral load). There was a statistically significant trend towards increasing D-dimer levels with rising levels of HIV-1 viral load. D-dimer was then correlated with IL-27 levels and a Spearman r showed no statistically significant correlation (Panel B). In Panel C, CRP levels were plotted for each group of patients and there was a trend towards increasing levels with rising HIV-1 viral loads. When IL-27 was plotted against CRP (Panel D), there was no significant correlation noted between these two parameters.

Persistent immune activation in HIV-1 infected patients has emerged as an important predictor of morbidity and mortality [13]. D-dimer and CRP levels were used to measure immune activation and were measured in all patients in this study. Both D-dimer (Figure 2A) and CRP (Figure 2C) were found to be associated with increasing HIV-1 viral load. There was a small but significant correlation observed between IL-27 and D-dimer levels (Spearman r = 0.09, p = 0.03) (Figure 2B), but no correlation was seen with CRP levels (Figure 2D, Spearman r = 0.03, NS). Whilst this is the first study to look at correlations of IL-27 and CRP in HIV-1 infected patients, studies in rheumatoid arthritis (RA) have also failed to show associations between IL-27 and CRP levels [14]. Whilst there was a small correlation noted between IL-27 and D-dimer, larger studies will have to be carried out to verify the strength of association. Higher levels of D-dimer have previously been shown to be associated with an increase in all-cause mortality in patients with HIV-1 infection who have interrupted therapy with ART [15]. In current study, we demonstrated that increasing viral load was associated with higher levels of sCD163 and D-dimer; however, there was no association noted between HIV-1 viral load and IL-27 levels. The small but significant correlations seen between IL-27 and D-dimer and IL-27 and sCD163 suggest a more complex interplay between these diverse markers of the immune system than just simply reflecting correlations related to exposure to the virus.

**Figure 3:**
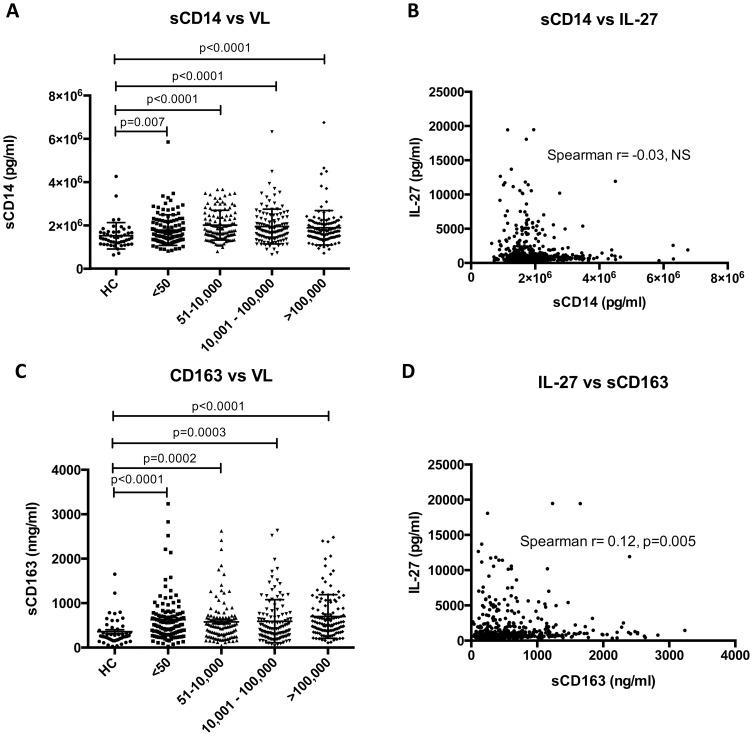
Correlation of sCD14 and sCD163 with plasma IL-27 levels. Plasma sCD14 levels, like the other markers of immune activation, were elevated in HIV-1 viremic patients compared to healthy controls (Panel A). Levels of sCD14 and plasma IL-27 levels were plotted (Panel B) but no correlation was observed. Plasma sCD163 levels were elevated in HIV-1 viremic patients compared to healthy controls (Panel C), with a trend towards higher levels of sCD14 with higher HIV-1 viral loads. Levels of sCD163 and plasma IL-27 levels were plotted (Panel B) and, unlike the other markers measured, there was a significant Spearman r noted (Spearman r = 0.12, p = 0.005) (Panel D).

Markers of monocyte activation have also been shown to correlate with HIV-1 disease progression in recent years, particularly soluble CD14 (sCD14) and soluble CD163 (sCD163). While translocation of microbial products (such as lipopolysaccharide or LPS) has been postulated to play an important role in HIV-1 pathogenesis through systemic immune activation [16] it is also possible that these changes may be due to activation of monocytes/macrophages by other stimuli. In this regard while sCD14 has been shown to be an independent predictor of mortality in HIV-1 infection this is not the case for LPS [17]. Toll-like receptor activation of macrophages and monocytes leads to shedding of sCD163, which functions as an innate immune receptor for bacteria. sCD163 levels have been shown to correlate with HIV-1 disease activity, so that they remain high in untreated patients with high HIV-1 RNA levels, and are lower in patients on ART [18]. We measured both of these parameters in this cohort of patients. sCD14 levels were shown to be significantly higher in patients with HIV-1 infection compared to uninfected controls (Fig 3A) but there was no correlation noted between IL-27 levels and sCD14 levels (Fig 3B). sCD163 was also significantly higher in patients with HIV-1 infection compared to healthy controls (Fig 3C). sCD163 levels, in contrast to sCD14, were found to significantly correlate with IL-27 levels (Spearman r = 0.12, p = 0.005) (Fig 3D). Although both sCD14 and sCD163 have been postulated as markers of monocyte activation, sCD14 is produced by both monocytes [19] and liver [20], whilst CD163 is more specifically expressed by monocytes and macrophages [21]. IL-27, likewise, is also mainly thought of as a cytokine secreted by activated monocytes/macrophages and the correlation between IL-27 and sCD163 may reflect specific monocyte/macrophage activation occurring within these cells.

In summary, we were not able to show any statistically significant differences in IL-27 levels in patients with HIV-1 infection compared to uninfected controls. In addition, there were no significant correlations observed between IL-27 and HIV-1 viral load and between IL-27 and CD4+ T cell counts. In analyzing IL-27 results in patients receiving ART, there was a significantly lower level of IL-27 in HIV+ patients receiving ART with viral loads of <50 copies/ml (compared to those not on treatment), which was not observed in patients on or off ART in the other viral load groups. This study represents the largest single center study examining the plasma level of IL-27 in HIV-1 infected individuals. Importantly, this study contrasts the results of two smaller studies, which suggested that there were possible relationships between HIV-1 infection and IL-27 levels [11], [12]. The positive correlation noted between IL-27 levels and D-dimer will need to be verified in larger studies but suggests that there may be a weak association with some markers of systemic inflammation. A correlation noted between sCD163 and IL-27 levels may reflect that both these markers are secreted by activated monocytes/macrophages. A study of all three biomarkers (IL-27, sCD163 and D-dimer) in HIV-1 infection may be worthwhile to see if the combination of markers provides a better prognostic model for predicting mortality and morbidity in these patients.
